# Hepatitis C Virus Infection Among non-IDU HIV-Infected and Uninfected Men who Have Sex with Men

**DOI:** 10.4084/MJHID.2011.058

**Published:** 2011-11-28

**Authors:** Massimo Giuliani, Lorenzo Nosotti, Alessandra Latini, Concetta Mirisola, Fulvia Pimpinelli, Sabrina Volpi, Fabrizio Ensoli, Gianpaolo Impara, Guido Palamara

**Affiliations:** 1 U.O.C. di Dermatologia Infettiva, San Gallicano Dermatological Institute (IRCCS), Rome; 2 National Institute for Health, Migration and Poverty (NIHMP), Rome; 3 Laboratorio di Patologia Clinica e Microbiologia, San Gallicano Dermatological Institute (IRCCS), Rome, Italy; 4 Dipartimento di Malattie Infettive, Parassitarie e Immunomediate (MIPI), Istituto Superiore di Sanità, Rome, Italy

**Dear Editor,**

Approximately one-third of the estimated 40 million people infected with HIV-1 worldwide, suffer from chronic hepatitis C virus (HCV) infection.^1^ In the Mediterranean countries, hepatitis C virus infection affects nearly 45% of HIV-1 infected individuals, consistently with the high proportion of patients with a history of intravenous drug use and who are exposed to the two viruses by parenteral route.^2^

Even in association with HIV-infection, HCV infection is rarely transmitted through sexual intercourse due to the lower efficiency of transmission by mucosal exposure with respect to the blood-borne one. Thus, the incidence and prevalence of HCV infection are far lower among the non-intravenous drug users (IDU) at risk of sexually transmitted infections (STI).

However, after 2000, several outbreaks of Hepatitis C virus infections have been observed in northern Europe and in the U.S.A. among non-IDU men who have sex with men (MSM), mostly HIV-infected.^3^–^5^ Epidemiologic investigations of clusters have shown that HCV infection was associated with high-risk sexual practices such as having a high number of partners, engaging in group sex, having a traumatic intercourse, use of rectal enema.

In 1997, our group has already reported a higher incidence of HCV infection among HIV co-infected MSM and suggested an increased risk of HCV among non-IDU immunosupressed MSM.^6^ Recently a study suggest that HCV-RNA can be found in seminal plasma of HIV infected individuals.^7^

Thus, we report the preliminary results from a HCV seroprevalence study conducted on consecutive HIV-infected and uninfected MSM, , who did not have a history of intravenous drug use, attending the Sexually Transmitted Infections (STI) Centre of the San Gallicano Dermatological Institute of Rome.

From January 2008 to December 2009, 203 non-IDU HIV-infected and 260 non IDU HIV-uninfected MSM were screened for antibodies against HCV. Median age was, 32 years (IQR=28–56) and 29 years (IQR=24–55) in the HIV-infected and the HIV-uninfected individuals, respectively. Fifty tree (26.1%) of the HIV-infected and forty four (16.9%) of the HIV-uninfected were non italian males.

The prevalence of HCV infection was 2.46% (95% CI:0.80–5.65) and 1.54 (95% CI:0.42–3.89) among the HIV-infected and HIV-uninfected, respectively. All HCV infected individuals were Italian MSM. Not significant statistical difference was observed between the two HCV prevalence rates (COR=1.62, 95% CI: 0.34–8.25).

Our data showed that the prevalence of HCV infection among MSM is higher than that observed in unselected MSM in England,^8^ but much far lower than that reported in HIV-infected MSM in the U.S.A, Australia and Holland.^9^–^12^

Two hypotheses may be taken in account to explain the lower prevalence rates observed in our seroprevalence study. The MSMs participating in our study could have less sexual contacts with IDU-MSMs than other gay community residents in other western countries. The non-IDU MSM recruited in this study could have a lower frequency of at-risk sexual practices for HCV than the non-IDU MSM enrolled in other studies.

Additional behavioral and phylogenetic investigations are needed to confirm these hypotheses. In particular, accurate behavioral investigation should be conducted in all HCV MSM patients, who deny any parental exposure, to better define the role of sexual exposure in the acquisition of infection. Moreover, phylogenetic approach to study HCV infections among population at risk for STI could reveal specific transmission networks and the comparison with genotyping profiles from IDU-MSM could confirm different characteristics of the transmission pathways.
